# The correlated factors of anxiety and depression among Chinese hospital staff during the COVID-19 local outbreak

**DOI:** 10.1097/MD.0000000000040190

**Published:** 2024-10-25

**Authors:** Guomiao Li, Chun Wei, Kai Fang, Hui Jiang, Quanwei Liu, Jiang Ou

**Affiliations:** a Department of Cancer Center, Second People’s Hospital of Neijiang, Neijiang, Sichuan, China; b Southwest Medical University, Luzhou, Sichuan, China; c The Affiliated Hospital of Southwest Medical University, Luzhou, Sichuan, China.

**Keywords:** anxiety, COVID-19, depression, hospital staff, mental health

## Abstract

Hospital staff in the COVID-19 local outbreak were facing different situations, their mental status and influencing factors were also different. The aim of this study is to investigate the anxiety and depression of hospital staff and its potential influence factors during the COVID-19 local outbreak. This was a cross-sectional survey based on a hospital with a local outbreak of COVID-19. We collected the demographic characteristics, COVID-19-related issues, self-rating anxiety scale (SAS), and self-rating depression scale (SDS) of participants through an anonymous questionnaire. Factors associated with anxiety and depression were explored through univariate and multivariate analyses. We also constructed nomograms and calibration curves to predict the probability of anxiety and depression. A total of 800 people completed the questionnaire. 239 (29.9%) of them were doctors, 249 (31.1%) of them were nurses and 312 (39.0%) of them were others. There were 173 (21.6%) cases had anxiety, including 36 (20.8%) doctors, 76 (43.9%) nurses and 61 (35.3%) from other occupations and 281 (35.1%) cases had depression, including 64 (22.8%) doctors, 101 (35.9%) nurses, and 116 (41.3%) from other occupations. Nurses had higher SAS and SDS score than doctors and others (F = 17.856, *P* < .001 and F = 14.376, *P* < .001). In addition, multivariate analysis found that occupation, education level, health condition, and reduced sleep were significant influences on anxiety and depression. At the same time, reduced income was also significantly associated with anxiety. During the local outbreak of COVID-19, hospital staff still had varying degrees anxiety and depression. Occupation, education level, health condition and reduced sleep were both significant influencing factors for anxiety and depression. The mental state of hospital staff, including nonmedical-related staff should still be taken seriously.

## 1. Introduction

The emergence of coronavirus disease 2019 (COVID-19) at the end of December 2019 triggered a global pandemic within a few months, posing a huge threat to the health of people worldwide.^[1,2]^ As of November 24, 2022, the World Health Organization has reported 635,709,158 confirmed cases of COVID-19 and 6,603,803 deaths, and 9,523,014 cases of COVID-19 were diagnosed in China, and 29,889 cases of death.^[3]^

Subsequently, due to the timely and strict epidemic prevention policy, COVID-19 in China tended to be stable. However, COVID-19 began to appear at different times and locations, China entered a period of the local outbreak.^[4,5]^ So far, COVID-19 in China mainly includes the national epidemic period, the stabilization period, and the local outbreak period. Although the transmission ability of new COVID-19 variants has increased during the local outbreak, the increase in vaccination rates has made COVID-19 a progressively lesser health threat.^[6–10]^ In this background, some East Asian countries such as South Korea and Japan have relaxed epidemic prevention and control measures to varying degrees, while China still maintains a strict COVID-19 prevention and control policy.^[11]^ The local outbreak period plays an extremely critical role in the overall prevention and control of the epidemic. The improvement of people’s awareness of epidemic prevention and the rates of vaccination have provided tremendous support to reduce the rate of severe cases and mortality during the fully open phase.

The hospital staff played an irreplaceable role in controlling COVID-19. Concern about the mental status of hospital staff has never stopped since the outbreak of COVID-19. In the early days of the pandemic, the lack of protective equipment and the heavy workload put hospital staff under tremendous psychological pressure.^[12–15]^ The study of COVID-19 in the stable period found that hospital staff still had different levels of anxiety and depression, which were often related to sex and occupation. To be specific, women and nurses often had more serious mental problems.^[16]^ In addition, a study of different periods of COVID-19 found that the anxiety and depression of hospital staff in the local outbreak period was significantly higher than those in other stages.^[17]^ This study focused on the mental status of hospital staff during the local outbreak period and explored the related factors which different with other periods leading to anxiety and depression. At the same time, we also analyzed other occupations working in hospitals during the local outbreak, including cleaners, security, medical technicians, and administrative staff, whose were also an important force to control the epidemic. In addition, we first constructed the nomograms to predict the probability of anxiety and depression for hospital staff.

## 2. Methods

### 2.1. Participants

This is a cross-sectional survey conducted within the Second People’s Hospital of Neijiang, Neijiang City, Sichuan Province, China, from September 16 to 27, 2022. The start of the current round of local outbreak was announced by the appearance of new COVID-19 cases in Neijiang on August 28, 2022. Subsequently, the number of new cases of COVID-19 continued to increase and reached its peak on September 15. After September 28, there were no new cases in Neijiang, which means the end of the local outbreak of COVID-19 that lasted for 1 month. The Second People’s Hospital of Neijiang has been under closed control and maintained normal medical activities during this COVID-19 epidemic. Considering the convenience of electronic questionnaires, we conducted an anonymous questionnaire survey of hospital staff using a professional questionnaire platform provided by www.wjx.cn. The questionnaire included the demographic characteristics, COVID-19-related issues, self-rating anxiety scale (SAS), and self-rating depression scale (SDS). In our institution, there were 480 doctors, 663 nurses, and about 700 other occupations. We sent out a wide range of surveys and eventually collected 800 completed questionnaires, including 239 doctors (51.9%), 249 nurses (37.6%), and 312 other occupations (44.6%). This study has been approved by the Ethics Committee of the Second People’s Hospital of Neijiang (Ethics number: 2022-005).

### 2.2. Evaluation of anxiety and depression

Anxiety and depression were assessed by SAS and SDS.^[18,19]^ Both SAS and SDS have 20 questions and 4 answers using a 4-point scale ranging from 1 (a little bit of the time) to 4 (most of the time). The standard score for SAS and SDS is the total score for all questions multiplied by 1.25. Anxiety levels were graded as follows: standard scores below 50 = normal, 50 to 59 = mild anxiety, 60 to 69 = moderate anxiety, and above 70 = severe anxiety. Depression level is graded as follows: standard score below 53 = normal, 53 to 62 = mild depression, 63 to 72 = moderate depression, 73 or more = severe depression. The Cronbach alpha coefficients for SAS and SDS were 0.845 and 0.868, demonstrating excellent reliability.

### 2.3. Statistical analysis

All statistical analysis was completed through the SPSS software 25.0 (Chicago) and R 4.1.3 (Vienna, Austria). Two-sided *P* values < .05 was considered as statistical differences. Continuous variables were expressed by means ± standard deviation, and differences between the 2 groups were compared by Student *t* test or the ANOVA test. Categorical variables were described by frequency rates and percentages, and differences between the 2 groups were analyzed by the chi-square test or Fisher exact test. Multivariate logistic regression analysis was used to find significant influencing factors on anxiety and depression. Finally, the nomograms and calibration curves based on the results of the multivariate analysis were constructed by the “rms” package to predict the probability of anxiety and depression.

## 3. Results

### 3.1. Characteristics of participants

A total of 800 people completed the questionnaire, including 249 (31.1%) men and 551 (68.9%) women. We categorized participants into doctors, nurses, and others based on their occupations in the hospital. The others include cleaners, security, medical technicians, and administrative staff. In this study, there were 239 doctors (29.9%), 249 nurses (31.1%), and 312 others (39.0%). The Chi-square test showed that occupation was related to sex, age, marital status, residence, expose to the COVID-19, concern about the source, fear of infection, work pressure, reduced sleep, SAS score, and SDS score (all *P* < .05). At the same time, the doctors were found to have higher education level (*P < *.001) by Fisher exact test. Anxiety was observed in 173 cases (21.6%), with 36 cases (20.8%) among doctors, 76 cases (43.9%) among nurses, and 61 cases (35.3%) among others. Furthermore, 281 cases (35.1%) exhibited symptoms of depression, with 64 cases (22.8%) among doctors, 101 cases (35.9%) among nurses, and 116 cases (41.3%) among those in other occupations. The detailed characteristics of all 800 cases grouped by occupation were displayed in Table 1.

**Table 1:** The characteristics of participants and outbreak-related issues.

Item	Level	Total	Doctors	Nurses	Others*	*P*
			239	249	312	
Sex (%)	Male	249 (31.1)	119 (49.8)	13 (5.2)	117 (31.1)	<.001
	Female	551 (68.9)	120 (50.2)	236 (94.8)	195 (62.5)	
Age (%)	<31	220 (27.4)	55 (23.0)	95 (38.2)	70 (22.4)	<.001
	31 to 40	326 (40.8)	100 (41.8)	116 (46.6)	110 (35.3)	
	>40	254 (31.8)	84 (35.1)	38 (15.3)	132 (42.3)	
Marital status (%)	Married	176 (21.9)	51 (21.3)	67 (26.9)	58 (18.6)	.023
	Non married	586 (73.3)	183 (76.6)	169 (67.9)	234 (75.0)	
	Divorced + Widow	38 (4.8)	5 (2.1)	13 (5.2)	20 (6.4)	
Education level (%)	Senior high school or lower	93 (11.6)	0 (0.0)	0 (0.0)	93 (29.8)	<.001
	Junior college or undergraduate	640 (80.0)	186 (77.8)	248 (99.6)	206 (66.0)	
	Master or above	67 (8.4)	53 (22.2)	1 (0.4)	13 (4.2)	
Residence (%)	Hospital	318 (39.8)	122 (51.0)	138 (55.4)	58 (18.6)	<.001
	Home	482 (60.2)	117 (49.0)	111 (44.6)	254 (81.4)	
Relationship with partners (%)	Well	698 (87.3)	212 (88.7)	218 (87.6)	268 (85.9)	.611
	Medium + Poor	102 (12.7)	27 (11.3)	31 (12.4)	44 (14.1)	
Relationship with parents (%)	Well	692 (86.5)	205 (85.8)	220 (88.4)	267 (85.6)	.586
	Medium + Poor	108 (13.5)	34 (14.2)	29 (11.6)	45 (14.4)	
Health condition (%)	Well	476 (59.5)	151 (63.2)	145 (58.2)	180 (57.7)	.381
	Medium + Poor	324 (40.5)	88 (36.8)	104 (41.8)	132 (42.3)	
Expose to the COVID-19 (%)	Yes	132 (16.5)	41 (17.2)	63 (25.3)	28 (9.0)	<.001
	No	668 (83.5)	198 (82.8)	186 (74.7)	284 (91.0)	
Concern about the source (%)	Mild	121 (15.1)	52 (21.8)	24 (9.6)	45 (14.4)	.001
	Moderate	510 (63.8)	149 (62.3)	157 (63.1)	204 (65.4)	
	Severe	169 (21.1)	38 (15.9)	68 (27.3)	63 (20.2)	
Fear of infection (%)	Mild	144 (17.9)	60 (25.1)	37 (14.9)	47 (15.1)	<.001
	Moderate	510 (63.8)	154 (64.4)	161 (64.7)	195 (62.5)	
	Severe	146 (18.3)	25 (10.5)	51 (20.5)	70 (22.4)	
Work pressure (%)	Mild	157 (19.6)	48 (20.1)	37 (14.9)	72 (23.1)	.047
	Moderate	467 (58.4)	148 (61.9)	153 (61.4)	166 (53.2)	
	Severe	176 (22.0)	43 (18.0)	59 (23.7)	74 (23.7)	
Reduced income (%)	Yes	594 (74.2)	176 (73.6)	189 (75.9)	229 (73.4)	.771
	No	206 (25.8)	63 (26.4)	60 (24.1)	83 (26.6)	
Reduced sleep (%)	Yes	486 (60.7)	124 (51.9)	177 (71.1)	185 (59.3)	<.001
	No	314 (39.3)	115 (48.1)	72 (28.9)	127 (40.7)	
Anxiety (%)	Normal	627 (78.4)	203 (84.9)	173 (69.5)	251 (80.4)	.001
	Mild	115 (14.4)	24 (10.0)	47 (18.9)	44 (14.1)	
	Moderate	40 (5.0)	7 (2.9)	22 (8.8)	11 (3.5)	
	Severe	18 (2.3)	5 (2.1)	7 (2.8)	6 (1.9)	
Depression (%)	Normal	519 (64.9)	175 (73.2)	148 (59.4)	196 (62.8)	.004
	Mild	168 (21.0)	36 (15.1)	53 (21.3)	79 (25.3)	
	Moderate	75 (9.4)	21 (8.8)	29 (11.6)	25 (8.0)	
	Severe	38 (4.8)	7 (2.9)	19 (7.6)	12 (3.8)	

* Others of occupation included caregivers, security, medical technicians, and administrative staff.

### 3.2. Univariate and multivariate analysis of anxiety and depression

To explore the independent predictive factors leading to anxiety and depression, we conducted logistic regression analyses for anxiety disorder (including mild, moderate, and severe anxiety) and depression disorder (including mild, moderate, and severe depression). We conducted univariate analyses of all 800 participants and found that anxiety was related to Marital status, education level, occupation, concern about the source, fear of infection, work pressure, relationship with partners, relationship with parents, health condition, reduced income, and reduced sleep (all *P* < .05). To avoid confounding factors and multicollinearity affecting the accuracy of the multivariate model, we calculated the Variance Inflation Factor (VIF) and Tolerance scores for factors that were significant in the univariate analysis. The results showed that the tolerance for all factors was >0.1, and the VIF was <10 for all, indicating no multicollinearity (Table 2). At the same time, we found that occupation (Risk ratio [RR] = 2.090, 95%Confidence interval [CI] = 1.380–3.165, *P* < .001), education level (RR = 0.555, 95% CI = 0.313–0.986, *P* = .044), health condition (RR = 2.046, 95% CI = 1.373–3.049, *P < *.001), reduced income (RR = 1.861, 95% CI = 1.050–3.301, *P* = .034), and reduced sleep (RR = 9.792, 95% CI = 5.086–18.853, *P* < .001) were independent predictors of anxiety (Table 3).Additionally, we discovered that depression was associated with occupation, educational level, work pressure, Relationship with partners, Relationship with parents, health condition, reduced income, and reduced sleep (All *P* < .05). Occupation (RR = 1.447, 95% CI = 1.018–2.057, *P* = .040), educational level (RR = 0.367, 95% CI = 0.221–0.609, *P* < .001), health condition (RR = 1.677, 95% CI = 1.195–1.678, *P* = .003), and reduced sleep (RR = 5.615, 95% CI = 3.797–8.304, *P < *.001) were also identified as independent predictive factors for depression (Table 4).

**Table 2:** The tolerance and VIF of all burnout related factors.

	Anxiety		Depression	
Items	Tolerance	VIF	Tolerance	VIF
Marital status	0.920	1.087		
Occupation	0.904	1.106	0.912	1.097
Education level	0.892	1.122	0.919	1.089
Concern about the source	0.730	1.369		
Fear of infection	0.767	1.303		
Work pressure	0.806	1.241	0.888	1.126
Relationship with partners	0.828	1.208	0.859	1.164
Relationship with parents	0.801	1.248	0.828	1.207
Health condition	0.855	1.169	0.864	1.158
Reduced income	0.878	1.138	0.880	1.136
Reduced sleep	0.817	1.224	0.836	1.196

VIF = variance inflation factor.

**Table 3:** The univariate and multivariate analyses of anxiety.

Items	Anxiety					
	Univariate analysis			Multivariate analysis		
	Risk ratio	95% CI	*P*	Risk ratio	95% CI	*P*
Sex						
Male	Ref					
Female	1.424	0.973 to 2.084	.069			
Age						
≤40	Ref					
>40	0.703	0.482 to 1.026	.068			
Marital status						
Married	Ref			Ref		
Others*	1.600	1.026 to 2.496	.038	1.652	0.990 to 2.757	.055
Occupation						
Doctors + others	Ref			Ref		
Nurses	2.056	1.452 to 2.911	<.001	2.090	1.380 to 3.165	<.001
Education level						
Senior high school or lower	Ref			Ref		
Others	0.599	0.371 to 0.967	.036	0.555	0.313 to 0.986	.044
Residence						
Hospital	Ref					
Home	0.827	0.588 to 1.163	.274			
Expose to the COVID-19						
No	Ref					
Yes	1.025	0.652 to 1.609	.916			
Concern about the source						
Mild	Ref			Ref		
Moderate + severe	3.133	1.645 to 5.969	.001	1.002	0.439 to 2.286	.996
Fear of infection						
Mild	Ref			Ref		
Moderate + severe	2.517	1.453 to 4.362	.001	1.450	0.728 to 2.887	.291
Work pressure						
Mild	Ref			Ref		
Moderate + severe	2.847	1.647 to 4.922	<.001	1.111	0.579 to 2.134	.751
Relationship with partners						
Well	Ref			Ref		
Medium/poor	2.120	1.353 to 3.322	.001	1.088	0.621 to 1.906	.769
Relationship with parents						
Well	Ref			Ref		
Medium/poor	2.026	1.302 to 3.150	.002	1.388	0.796 to 2.420	.248
Health condition						
Well	Ref			Ref		
Medium/poor	2.621	1.857 to 3.698	<.001	2.046	1.373 to 3.049	<.001
Reduced income						
No	Ref			Ref		
Yes	3.688	2.199 to 6.185	<.001	1.861	1.050 to 3.301	.034
Reduced sleep						
No	Ref			Ref		
Yes	13.773	7.332 to 25.872	<.001	9.792	5.086 to 18.853	<.001

* Others of marital status: non married + divorced + widow; others of education level: junior college or undergraduate + master or above; others of occupation include caregivers, security, medical technicians, and administrative staff.

**Table 4:** The univariate and multivariate analyses of depression.

Items	Depression					
	Univariate analysis			Multivariate analysis		
	Risk ratio	95% CI	*P*	Risk ratio	95% CI	*P*
Sex						
Male	Ref					
Female	1.244	0.910 to 1.701	.171			
Age						
≤40	Ref					
>40	0.893	0.656 to 1.217	.474			
Marital status						
Married	Ref					
*Others	1.039	0.735 to 1.468	.830			
Occupation						
Doctors + others	Ref			Ref		
Nurses	1.456	1.072 to 1.976	.016	1.447	1.018 to 2.057	.040
Education level						
Senior high school or lower	Ref			Ref		
Others	0.429	0.277 to 0.664	<.001	0.367	0.221 to 0.609	<.001
Residence						
Hospital	Ref					
Home	0.949	0.709 to 1.271	.726			
Expose to the COVID-19						
No	Ref					
Yes	1.154	0.781 to 1.706	.471			
Concern about the source						
Mild	Ref					
Moderate + Severe	1.444	0.952 to 2.190	.084			
Fear of infection						
Mild	Ref					
Moderate + Severe	1.408	0.957 to 2.071	.082			
Work pressure						
Mild	Ref			Ref		
Moderate + severe	2.167	1.456 to 3.226	<.001	1.143	0.721 to 1.812	.569
Relationship with partners						
Well	Ref			Ref		
Medium/poor	2.054	1.352 to 3.122	.001	1.261	0.767 to 2.073	.360
Relationship with parents						
Well	Ref			Ref		
Medium/poor	2.239	1.486 to 3.373	<.001	1.574	0.960 to 2.581	.072
Health condition						
Well	Ref			Ref		
Medium/poor	2.216	1.654 to 2.971	<.001	1.677	1.195 to 2.354	.003
Reduced income						
No	Ref			Ref		
Yes	2.209	1.547 to 3.153	<.001	1.117	0.743 to 1.678	.595
Reduced sleep						
No	Ref			Ref		
Yes	6.654	4.631 to 9.560	<.001	5.615	3.797 to 8.304	<.001

* Others of marital status: non married + divorced + widow; others of education level: junior college or undergraduate + master or above; others of occupation include caregivers, security, medical technicians, and administrative staff.

### 3.3. Construction of nomograms to predict anxiety and depression

**Figure 1.**
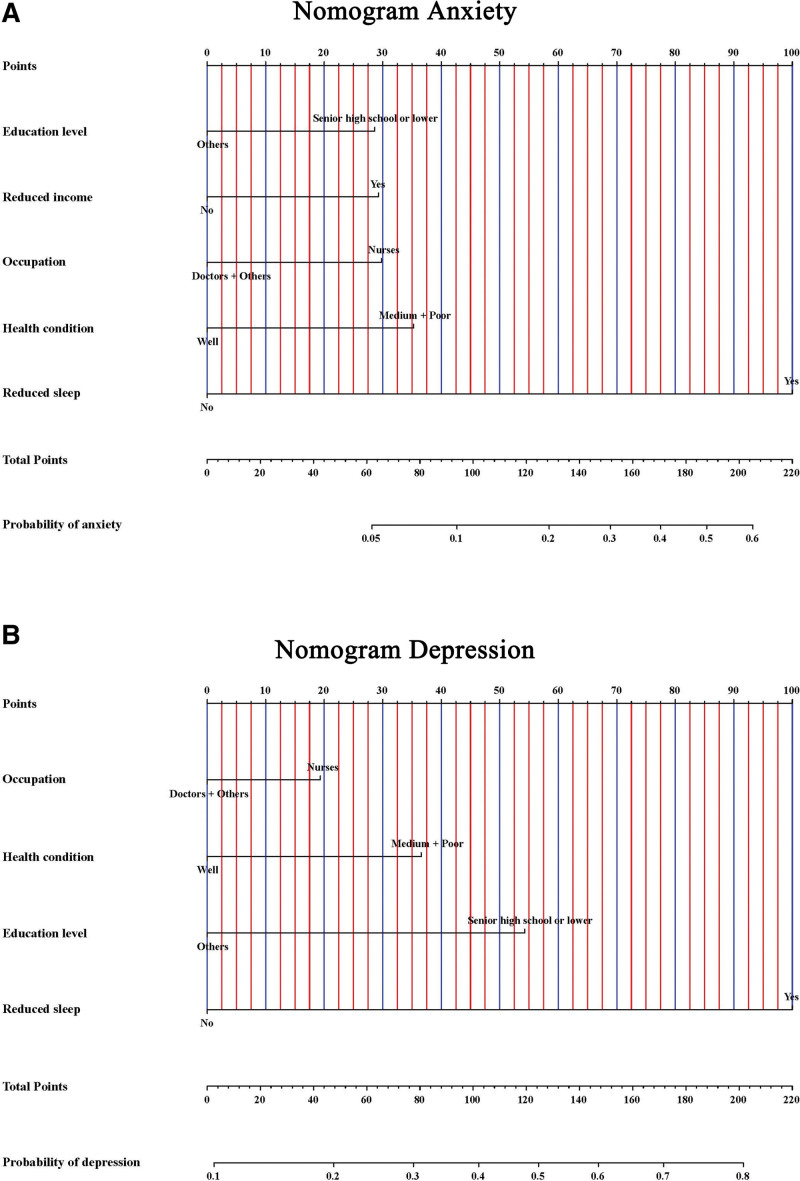
Nomograms for predicting anxiety (A) and depression (B).

**Figure 2.**
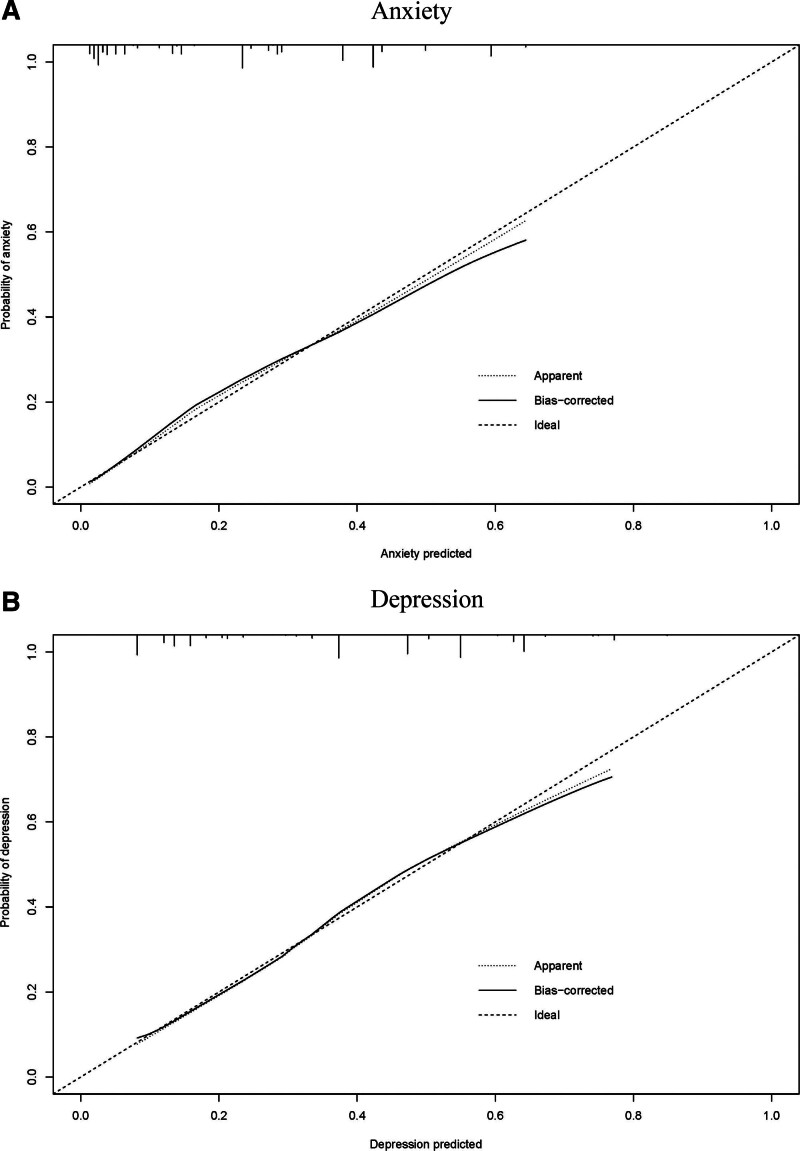
The calibration curve of nomograms for predicting anxiety (A) and depression (B).

We found that occupation, education level, health condition, and reduced sleep were both related to anxiety and depression. In addition, anxiety was also associated with reduced income. So, the nomograms to predict the probability of anxiety and depression based on the results of the multivariate analysis were established (Fig. 1A and B). The C-index for predicting the probability of anxiety and depression were 0.796 and 0.762, respectively. At the same time, the calibration curve of the nomograms for predicting anxiety and depression demonstrated good agreement between prediction and observation (Fig. 2A and B).

## 4. Discussion

COVID-19 local outbreak period is an important part of epidemic control in China. Further study for local outbreak period could help to understand the mental status of hospital staff and could also accumulate experience for possible public health emergencies in the future. This study investigated anxiety and depression among hospital staff and their influencing factors during the local outbreak of COVID-19 in China. We also studied the anxiety and depression of nonmedical-related staff who work in hospital. Finally, we established the nomograms for the first time to predict the probability of anxiety and depression of hospital staff.

At the beginning of the COVID-19 pandemic, the outbreak caused tremendous pressure among hospital staff, resulting in varying degrees of anxiety and depression.^[20–22]^ In 2020, Zhan et al studied the anxiety and depression status of 201 healthcare workers and found that the Incidence of anxiety and depression were 15.9% and 21.9%, respectively. Age and marital status were significant factors influencing anxiety and depression.^[23]^ Another cross-sectional survey collected SAS and SDS score from 3144 healthcare workers in 7 hospitals through questionnaires and found that out of 3144 participants, a total of 663 were in anxiety and 1381 were in depression, with prevalence rates of 21.1% and 43.9% for anxiety and depression, respectively. Multivariate analysis revealed that age remained a significant factor influencing anxiety and depression.^[24]^ A study by Xing et al came to similar conclusions. They collected demographic information as well as SAS and SDS score from 309 healthcare workers in a healthcare facility through an anonymous questionnaire and found that the prevalence of anxiety and depression were 20.1% and up to 56.0%, with more than half of the healthcare workers in depressed.^[25]^

In this study, the prevalence rates of anxiety and depression of all hospital staff were 21.6% and 35.1%. We analyzed different occupation and found that doctors, nurses, and others had varying degrees of anxiety and depression. In addition, nurses have higher SAS and SDS score and higher rates of anxiety and depression than doctors and others. In the further multivariate analysis, we found that occupation, education level, health condition reduced income, and reduced sleep were significant influencing factors for anxiety. In addition, occupation, education level, health condition, and reduced sleep were also significantly related to depression.

The prevalence rates of anxiety and depression of all hospital staff in this study were still comparable to the initial level of the epidemic. Salari et al collected the research on the mental status of hospital staff from December 2019 to June 2020 and found that the prevalence rates of anxiety and depression were 25.8% and 24.3% respectively.^[26]^ Another study from Luo et al integrated the results of 62 studies on the mental status of hospital staff and found that the rates of anxiety and depression were 33% and 28%.^[27]^ With the increasing experience of people in fighting against COVID-19 and the rising vaccination rate, the threat of COVID-19 to people’s health was decreasing. In addition, as early as the early days of COVID-19, the psychological state of hospital staff was given attention and hospitals had special departments to monitor and intervene in the psychological problems of hospital staff. Under these circumstances, hospital staff continued to experience varying degrees of anxiety and depression. This further illustrates the pressure that hospital staff was undergoing from multiple sources during the local outbreak. Psychological support for hospital staff was still necessary during the local outbreak of COVID-19.

Previous studies have found that nurses were more likely to suffer from anxiety and depression than others, and our study was the same.^[28–30]^ During the local outbreak of COVID-19, hospitals in China had strict prevention policies in place, including multiple nucleic acid tests and restrictions on the movement in hospital of patients. Nurses often participate in the implementation of these policies. These not only lead to an increase in nurses’ workload but may also trigger conflicts between nurses and patients, all of which expose nurses to more serious mental problems. In addition, correlation analysis found that occupation was associated with age, sex, education level, expose to the COVID-19, and reduced sleep. Many studies have shown that these were important factors of anxiety and depression, and they were also characteristics of most nurses.^[31–34]^ It is worth noting that other occupations of the hospital also had mental problems and were under great pressure from the COVID-19 epidemic prevention policy. However, psychological support for hospital staff often ignored these persons.

Lower education level and poor health condition had been found to be the significant contributors to anxiety and depression in many early studies.^[35–39]^ This situation did not change during the local outbreak of COVID-19. We also found that age and marital status were significant contributors to anxiety. Younger people have higher family and financial burdens, while unmarried people receive less family support. Concern about the source was also significantly associated with the occurrence of depression, suggesting that identifying the source of the current COVID-19 transmission could help reduce depression levels in hospital staff. Many studies have pointed out the huge impact of reduced sleep on mental status.^[40,41]^ Reduced sleep was the factor that had the greatest impact on anxiety and depression in our study. During the period of the COVID-19 local outbreak, many hospital staff had to live in the hospital for a long time to ensure the delivery of medical services. Strange living environments reduced the sleep time of hospital staff, leading to anxiety and depression.

This study showed that it is still necessary to provide psychological support to hospital staff, especially nurses, during the local outbreak of COVID-19. At the same time, the mental status of nonmedical-related staff in hospital should not be ignored. In addition, finding out the source of this round of epidemic and improving the sleeping conditions of hospital staff might help alleviate the pressure of the epidemic and improve the psychological state.

There were some unavoidable limitations of this study. First, this is a single-center cross-sectional survey that needs to be further validated by a multicenter study with a larger sample size. Second, data collection was voluntary through an anonymous questionnaire, and potential information bias that may exist must be considered. Finally, we were unable to cover all risk factors associated with COVID-19 in this investigation.

## 5. Conclusion

During the local outbreak of COVID-19, hospital staff still had varying degrees anxiety and depression. Age, marital status, concern about the source of COVID-19, occupation, education level, health condition and reduced sleep were both significant influencing factors for anxiety and depression. The mental state of hospital staff, including nonmedical-related staff should still be taken seriously.

## Author contributions

**Data curation:** Kai Fang, Hui Jiang.

**Funding acquisition:** Jiang Ou.

**Investigation:** Kai Fang, Hui Jiang.

**Methodology:** Quanwei Liu.

**Project administration:** Jiang Ou.

**Resources:** Jiang Ou.

**Supervision:** Quanwei Liu.

**Writing – original draft:** Guomiao Li, Chun Wei.

**Writing – review & editing:** Guomiao Li, Chun Wei.
